# SELDI-TOF-MS determination of hepcidin in clinical samples using stable isotope labelled hepcidin as an internal standard

**DOI:** 10.1186/1477-5956-6-28

**Published:** 2008-10-14

**Authors:** Douglas G Ward, Keith Roberts, Paul Stonelake, Patrick Goon, Cleidiane G Zampronio, Ashley Martin, Philip J Johnson, Tariq Iqbal, Chris Tselepis

**Affiliations:** 1 School of Cancer Studies, College of Medical and Dental Sciences, University of Birmingham, Birmingham, B15 2TT, UK; 2 Russells Hall Hospital, Pensnett Road, Dudley, Stourbridge, DY1 2HQ, UK; 3 School of Biosciences, University of Birmingham, Birmingham, B15 2TT, UK

## Abstract

**Background:**

Hepcidin is a 25-residue peptide hormone crucial to iron homeostasis. It is essential to measure the concentration of hepcidin in cells, tissues and body fluids to understand its mechanisms and roles in physiology and pathophysiology. With a mass of 2791 Da hepcidin is readily detectable by mass spectrometry and LC-ESI, MALDI and SELDI have been used to estimate systemic hepcidin concentrations by analysing serum or urine. However, peak heights in mass spectra may not always reflect concentrations in samples due to competition during binding steps and variations in ionisation efficiency. Thus the purpose of this study was to develop a robust assay for measuring hepcidin using a stable isotope labelled hepcidin spiking approach in conjunction with SELDI-TOF-MS.

**Results:**

We synthesised and re-folded hepcidin labelled with ^13^C/^15^N phenylalanine at position 9 to generate an internal standard for mass spectrometry experiments. This labelled hepcidin is 10 Daltons heavier than the endogenous peptides and does not overlap with the isotopic envelope of the endogenous hepcidin or other common peaks in human serum or urine mass spectra and can be distinguished in low resolution mass spectrometers. We report the validation of adding labelled hepcidin into serum followed by SELDI analysis to generate an improved assay for hepcidin.

**Conclusion:**

We demonstrate that without utilising a spiking approach the hepcidin peak height in SELDI spectra gives a good indication of hepcidin concentration. However, a stable isotope labelled hepcidin spiking approach provides a more robust assay, measures the absolute concentration of hepcidin and should facilitate inter-laboratory hepcidin comparisons.

## Background

Hepcidin, a 25-residue peptide hormone, is a key regulator of iron homeostasis [1-3]. It is produced by hepatocytes and to a lesser extent by macrophages, bacteria-activated neutrophils and colorectal cancer cells [2-5]. The major stimuli for hepcidin expression include iron excess, inflammation and infection. Hepcidin exerts its biological effect at the level of cellular iron export by binding to and causing the internalisation and degradation of ferroportin [6]. Thus in macrophages; the major cell type responsible for iron recycling, the iron becomes trapped resulting in an anaemia which in the context of inflammation and infection is characterised as the anaemia of chronic disease [7].

There has been intense research into how hepcidin is regulated and its role in pathologies including haematological disorders, liver disease and carcinogenesis [1,5,8]. The method most commonly employed for measuring hepcidin in serum and urine is surface enhanced laser desorption/ionisation time-of-flight mass spectrometry (SELDI) [5,9-12]. SELDI offers facile high-throughput sample preparation via on-chip retentate chromatography with hepcidin binding to NP20, CM10 and IMAC surfaces (normal-phase silica, cation exchange or immobilised metal ion chromatography respectively). It is assumed the height of the SELDI peak at m/z 2791 is related to hepcidin concentration. However, although Tomosugi *et al *report a linear relationship between SELDI peak height and hepcidin concentration under ideal conditions [12] and Bozzini *et al *demonstrate a correlation between SELDI peak height and a dot-blot immunoassay [9], this may not be a valid assumption when comparing samples with variable proteomic backgrounds or using different instruments. Doubts have been raised about the reproducibility of SELDI data [13-15]. Recently Swinkels and coworkers [16,17] have used a truncated version of hepcidin (hepcidin-24) as an internal standard. Most recently, Ganz *et al *and Kobold *et al *have reported ELISA and LC-ESI-MS with a stable isotope labelled standard to quantitate hepcidin [18,19]. We now report the development of a simple alternative method to assay hepcidin in human serum combining the use of stable isotope labelled hepcidin and SELDI-TOF-MS.

## Methods

### Hepcidin synthesis and folding

Human hepcidin was synthesised with or without ^13^C/^15^N phenylalanine at position 9 (AltaBioscience, University of Birmingham). This was dissolved at 0.1 mg/ml in 6 M urea, 30 mM MOPS (pH 7.0) and incubated overnight at room temperature with stirring. The folded hepcidin was purified by C18 RP-HPLC in 0.1% TFA/acetonitrile. Hepcidin concentrations were determined by BCA assay calibrated with bovine serum albumin (Pierce).

### Sample collection

Serum was collected from women attending routine breast clinics at Russell's Hall Hospital, Dudley, UK between 2005 and 2007 (LREC Ref 05/Q2709/48). All subjects gave informed consent prior to venipuncture. Venous blood was taken into serum collection tubes and allowed to clot at room temperature for 1–2 hours. Samples were then centrifuged for 10 min at 3000 g and the supernatant stored in aliquots at -80°C. The urine sample used in the experiment of Figure 7 was selected on the basis of hepcidin peak height from a previously analysed set of urine samples [20].

### SELDI

Sera were analysed on Cu^2+^-loaded IMAC30 ProteinChips using a PBS IIc time-of-flight mass spectrometer (BioRad). Sera were diluted 5-fold in 8 M urea, 1% CHAPS in binding buffer (0.5 M NaCl, 0.1 M sodium phosphate, pH 7.0) followed by a further 10-fold dilution in binding buffer and 100 μl applied to the chips. Following 30 minutes binding the chips were washed with binding buffer, rinsed with water, dried and 2 × 1 μl of 50% saturated sinapinic acid in 50% acetonitrile/0.5% TFA added. Spectra were collected over m/z 0–20,000 focussed at m/z 2800 using a laser power of 165. Following mass calibration, total ion current normalisation and baseline subtraction the hepcidin peaks were manually picked and intensities (peak heights) extracted using ProteinChip software.

### Immunofluoresence

THP-1 cells were cultured in RPMI 1640 media containing 10% FCS, 2 mM L-glutamine, 1% penicillin/streptomycin. To promote differentiation/activation cells were incubated with 16 nM phorbol-12-myristate-13-acetate for 48 hours. Cells were then treated for 24 hrs with/without 0.5 μM folded or reduced labelled hepcidin (reduced with 2 mM tris(2-carboxyethyl)phosphinehydrochloride). Cells were then fixed, blocked and incubated for 1 hr with an anti-ferroportin rabbit polyclonal (1:100, clone 3566, this antibody was raised against the oligopeptide CGKQLTSPKDTEPKPLEGTH corresponding to amino acids 247–264 of murine ferroportin as previously described [21]). Cells were then labelled with FITC goat anti-rabbit (Jackson Immunoresearch, 1:500), washed and visualised.

### NMR spectroscopy

Spectra were acquired at 25°C on a Varian INOVA-800 spectrometer equipped with a cryoprobe as previously reported [22]. Briefly, hepcidin (0.5 mg/ml) was prepared in 50 mM phosphate buffer (pH 4.0) containing 120 mM KCl in 90% H_2_O/10% D_2_O and ^1^H-^1^H through space correlations obtained using standard homonuclear 2D NOESY experiments with a 150-ms mixing time.

### Statistics and analysis of reproducibility

Statistical significance was calculated using an unpaired two tailed Student's t test. Trend lines were fitted using standard least squares linear regression. Reproducibility was estimated by analysing 2 serum samples spiked with 200 ng hepcidin/ml in quadruplicate on ProteinChip arrays on 5 successive days. The intra-assay coefficients of variation (CV (mean/standard deviation)) were calculated from the 4 replicates of each sample on each ProteinChip array and the inter-assay CVs by comparing across days.

## Results

### Hepcidin Folding

**Figure 1:**
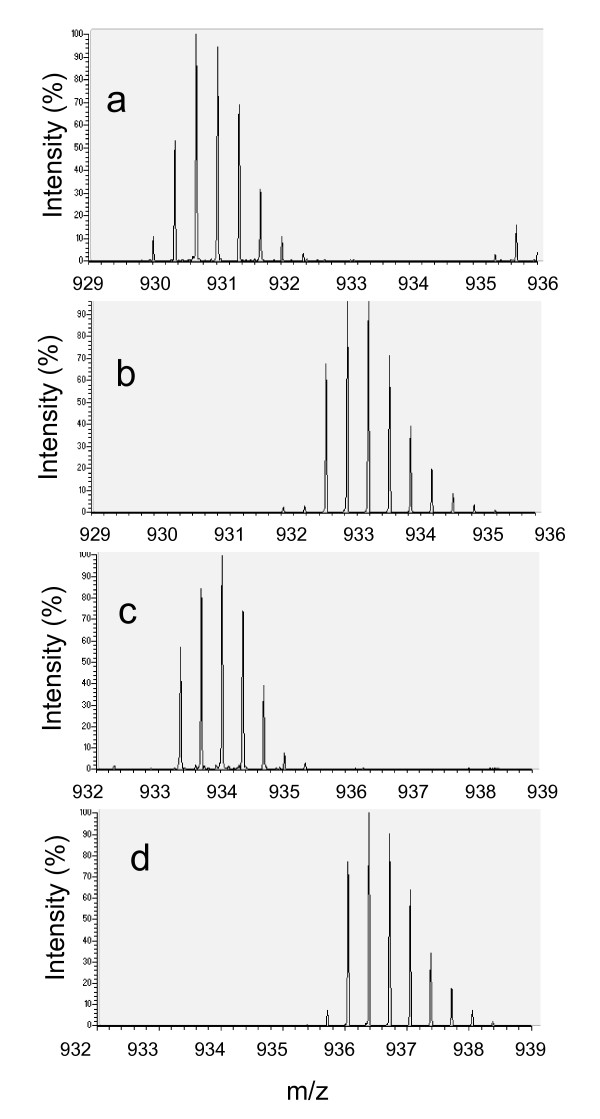
**FTICR mass spectra of folded and reduced synthetic hepcidin-25**. We show the 3^+^ion of unlabelled folded hepcidin 25 (a), reduced unlabelled hepcidin (b), folded labelled hepcidin (c) and reduced labelled hepcidin (d). Spectra were acquired by direct infusion in 0.1% formic acid/50% acetonitrile into a ThermoFinnigan LTQ-FT. Reduced samples were treated with DTT, zip-tipped and kept at low pH prior to analysis.

During folding 4 disulphide bridges form with the loss of 8 hydrogen atoms resulting in an 8 Da decrease in molecular weight. We used ESI-FTICR mass spectrometry to detect this mass change. The 3^+ ^ions of the folded and reduced forms of unlabelled and labelled hepcidin are shown in Figure 1. The measured masses of the folded and reduced unlabelled hepcidin, 2787.033 and 2795.106 Da, agree with predicted values (2787.025 and 2795.088 respectively). The measured masses of the folded and reduced labelled hepcidin are 2797.053 and 2805.134 Da (predicted values 2797.053 and 2805.116).

**Figure 2:**
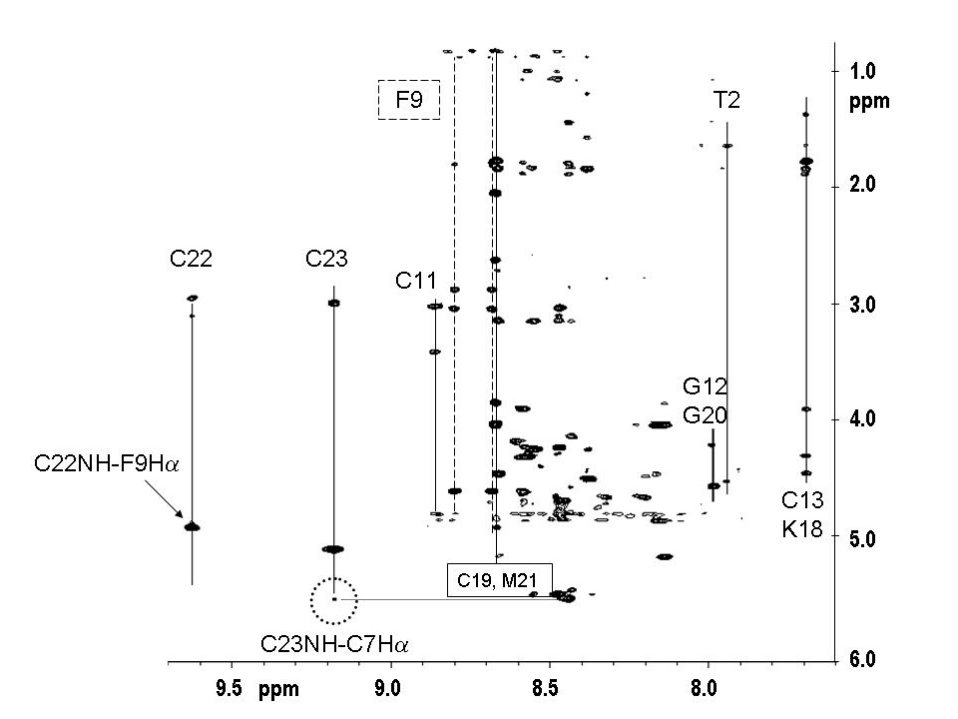
**NMR analysis of folded synthetic hepcidin**. Section of the ^1^H-^1^H NOESY spectrum showing crosspeaks associated with the backbone -NH and aromatic groups of hepcidin. Through space correlations of the -NH of several of the Cys residues are identified. Some of the ^1^H proximities prescriptive of the published fold of the human hepcidin molecule are circled.

We have used 2D proton NMR to characterize the folded structure of our synthetic hepcidin. Spatial proximities identified in the NOESY spectrum of hepcidin were used to deduce the correspondence between our folded hepcidin and the published structure (Figure 2 and reference [22]). Homology of folding can be recognised by spectral shift pattern matching in combination with observed long-range NOE and it is this dual approach, focussing on specific residues involved in critical contacts within the hepcidin molecule fold that we used to confirm the architecture of our synthetic hepcidin. Consistent with the homology of S-S linkages, the pattern and dispersion of chemical shifts of backbone -NH (and cysteine -CaH, 4.9–5.5 ppm – data not shown) obtained for the synthetic hepcidin matches the shift and intramolecular ^1^H-^1^H distance fingerprint of the published human hepcidin structure [22].

**Figure 3:**
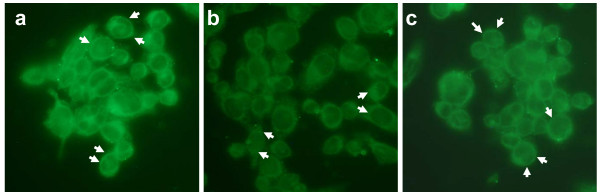
**The effect of stable isotope labelled hepcidin on ferroportin immunolocalisation**. Ferroportin immunoreactivity on THP-1 cells incubated without labelled hepcidin (a), with folded labelled hepcidin (b) and reduced labelled hepcidin (c). Arrows denote areas of positivity.

The biological activity of the refolded labelled hepcidin was tested by measuring ferroportin internalisation by immunofluorescence. In the absence of hepcidin ferroportin was predominantly localised on the cell border of THP-1 cells (Figure 3a). Addition of labelled hepcidin caused a re-localisation of ferroportin immunoreactivity to the cytoplasm (Figure 3b) whereas reduced (unfolded) hepcidin had a less marked effect (Figure 3c).

### Hepcidin Measurement by SELDI

**Figure 4:**
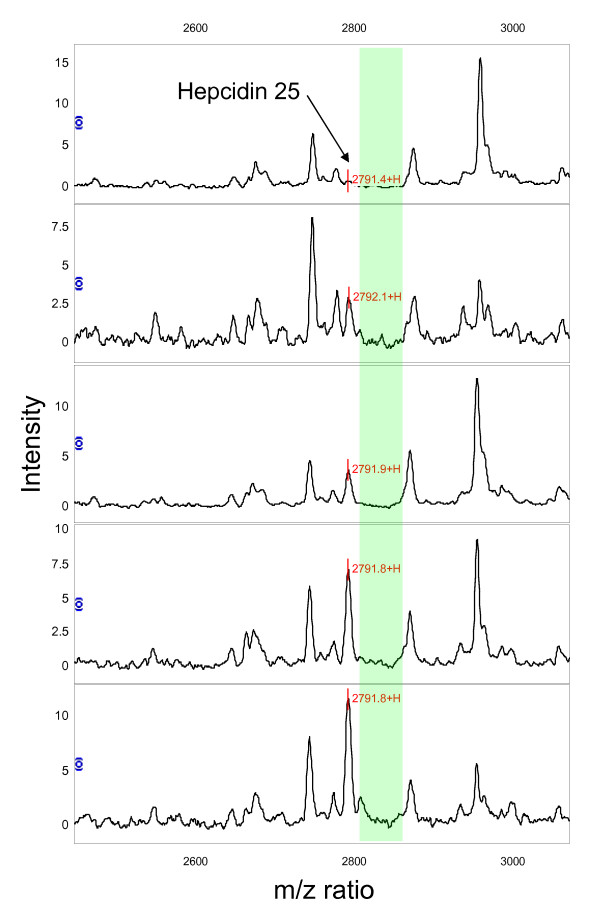
**SELDI spectra of human sera**. IMAC spectra of 5 serum samples with increasing hepcidin peak heights (top to bottom). Green shading denotes a region of spectrum close to the endogenous hepcidin peak suitable for design and inclusion of an internal standard.

Spectra obtained from the sera of 5 breast cancer patients using Cu^2+ ^loaded IMAC arrays are shown in Figure 4. A peak with m/z 2791 corresponding to hepcidin-25 can be seen in most of the spectra. There is little evidence of hepcidin-20 or the oxidised form of hepcidin-25 in this set of sera. The background proteome is variable, however, there is a region of low background to the right of the hepcidin-25 peak (m/z 2800–2850) offering a window in which to introduce an internal standard.

### Hepcidin Assay Validation

**Figure 5:**
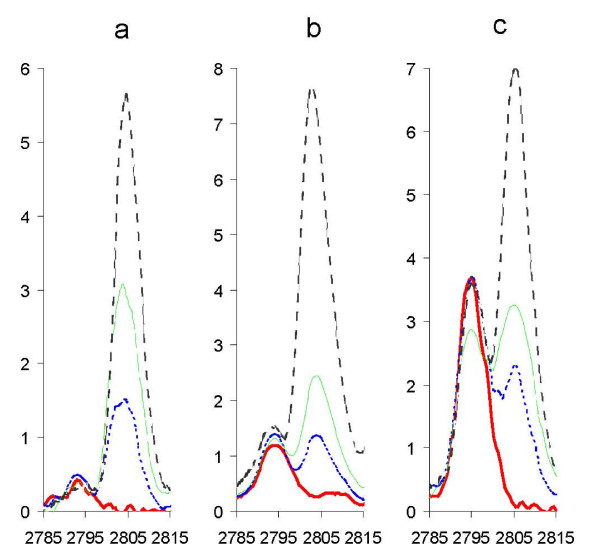
**Titration of labelled hepcidin into serum samples**. Labelled hepcidin was spiked into sera with low (a), medium (b) and high (c) levels of endogenous hepcidin (estimated from SELDI spectra). The endogenous hepcidin produces a peak at m/z 2791 and the labelled hepcidin a peak at m/z 2801. Labelled hepcidin concentrations are: bold red line: 0 ng/ml, dashed blue line: 100 ng/ml, dashed green line (200 ng/ml), dashed black line: 400 ng/ml.

**Figure 6:**
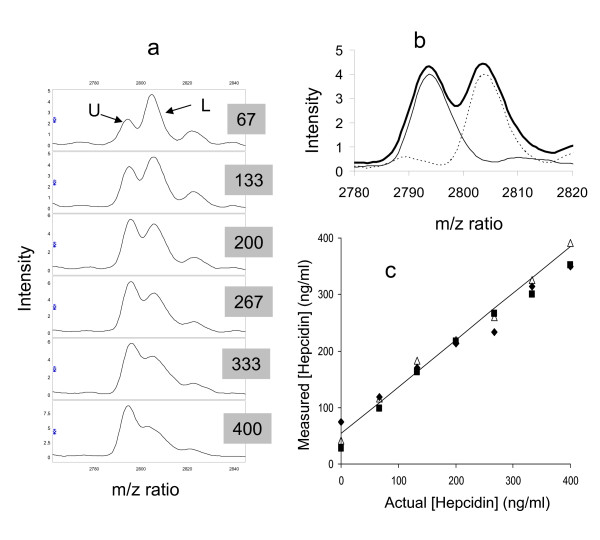
**SELDI analysis of hepcidin mixes**. a) spectra of serum with 200 ng/ml labelled hepcidin (L) added in addition to 67–400 ng/ml unlabelled hepcidin (U) as indicated in the grey boxes. b) spectra of labelled (dotted line) and unlabelled (solid line) hepcidin at 200 ng/ml in serum and the sum of the 2 spectra (bold line). c) shows the concentration of unlabelled hepcidin calculated from the peak height ratio plotted against the actual concentration in the experiment of Figure 6a and two independent replicates in different sera. The trend line represents a least-squares linear regression (r^2 ^= 0.990).

Labelled hepcidin was titrated into serum (0–400 ng hepcidin/ml) without influencing the intensity of the endogenous hepcidin SELDI peak (Figure 5). In experiments adding 0–50 ng hepcidin/ml to serum, the lowest concentration of labelled hepcidin generating a reliable peak was ~10 ng/ml indicating that this would also be the limit of detection of endogenous hepcidin in serum by this method (data not shown). A working concentration of 200 ng labelled hepcidin/ml was adopted as this produces a robust peak similar to the highest endogenous peak observed. We then added 0–400 ng/ml unlabelled and 200 ng/ml labelled hepcidin to a serum sample devoid of endogenous hepcidin to test whether the peak height ratio is representative of the concentration (Figure 6a). Although the edges of the peaks overlap, when the spectra of 200 ng/ml labelled and unlabelled hepcidin are superimposed neither peak contributes significantly to the peak height of the other (Figure 6b). Plotting unlabelled hepcidin concentrations calculated from peak height ratios against actual concentration (Figure 6c) reveals a strong correlation (the non-zero intercept is largely due to the endogenous hepcidin in the sera used and a slight downwards curvature is seen at very high hepcidin concentrations). This indicates that the peak height ratio produces a reliable estimate of hepcidin concentration.

**Figure 7:**
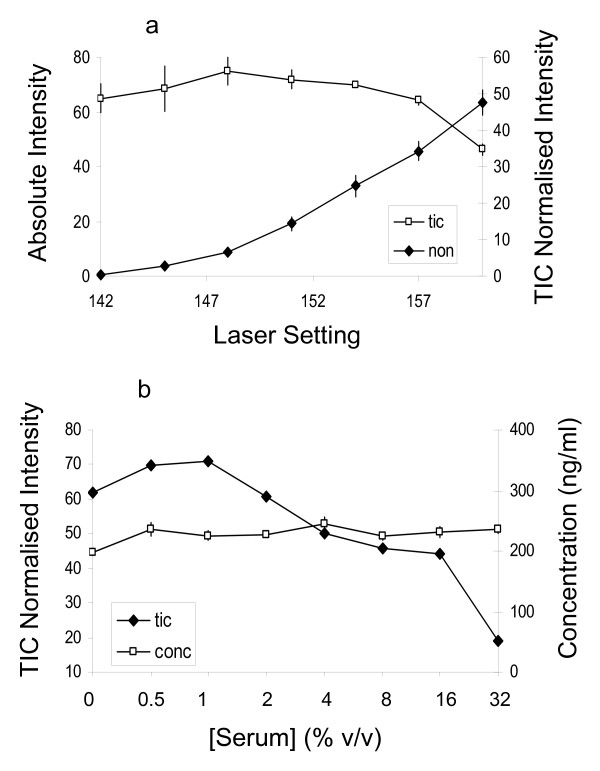
**Hepcidin analysis in urine under different conditions**. A urine sample was diluted to 20 μg protein/ml and spiked with 200 ng/ml labelled hepcidin and 0–32% (v/v) human serum devoid of hepcidin (as estimated from SELDI spectra – see the top spectrum in Figure 4). Panel a: endogenous hepcidin peak intensity with/without TIC-normalisation plotted against laser power. Filled symbols denote mean (± SEM) non-normalised intensity of all 8 samples at each laser setting (not [serum] dependent). Open symbols denote mean (± SEM) TIC-normalised intensity of the 4 samples with 0–32% serum added (the TIC normalised is [serum] dependent). Panel b plots the TIC-normalised hepcidin intensity and the concentration calculated from the peak ratio over a range of serum concentrations (constant laser power).

To assess the effect of background proteome and instrument variability we used the peak height ratio method to determine hepcidin in urine with varying amounts of irrelevant proteins added and at various laser settings (Figure 7). Urine with ~200 ng/ml endogenous hepcidin was spiked with 200 ng/ml labelled hepcidin and increasing amounts of a serum sample not containing hepcidin. The absolute height of the endogenous hepcidin peak was strongly dependent on laser power but not affected by the addition of serum (Figure 7a). Total ion current (TIC) normalisation overcame the dependence on laser power but was influenced by addition of serum (Figure 7b). The concentration of endogenous hepcidin calculated from the peak height ratio was not influenced by sample composition or instrument performance as shown in Figure 7b.

### Comparison with non-spiked SELDI data

**Figure 8:**
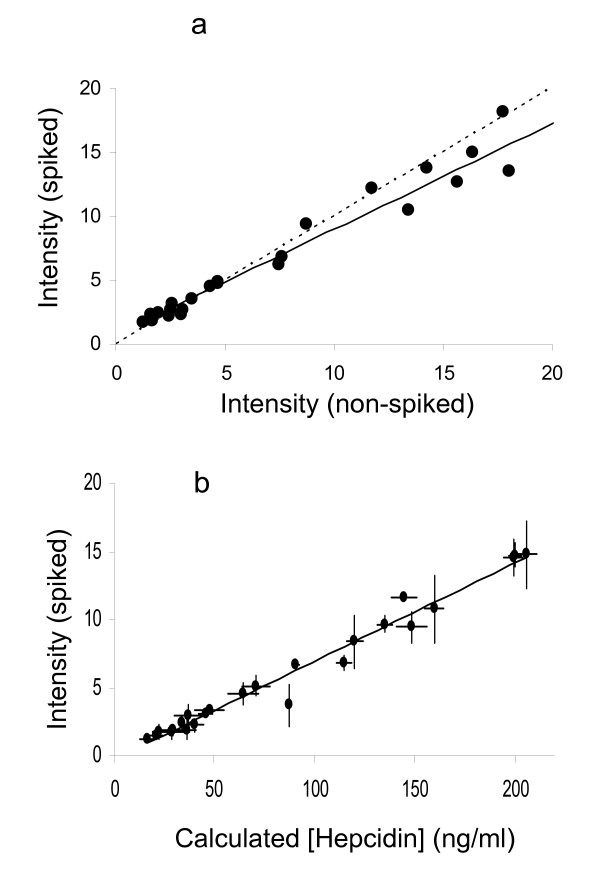
**Comparison of hepcidin concentration and SELDI peak height**. Two independent SELDI analyses of 24 serum samples are shown in Figure 8a. SELDI was performed in triplicate following spiking with 200 ng/ml labelled hepcidin and compared with SELDI without spiking (performed in duplicate). Symbols denote the means of experimental replicates, the least-squares linear regression (R^2 ^= 0.827) is shown as a solid line and the ideal 1:1 relationship as a dashed line. Figure 8b shows the relationship between endogenous hepcidin peak height and concentration determined from the peak height ratio in the spiked experiment. Symbols denote mean (± SEM) of experimental triplicates and the trend line least-squares linear regression (R^2 ^= 0.975).

We analysed 24 serum samples with a range of hepcidin peak heights with and without spiking with 200 ng/ml labelled hepcidin. The strong correlation between the endogenous hepcidin peak heights in the spiked and non-spiked assays (Figure 8a) re-emphasises that spiking has little effect on the endogenous hepcidin peak height and shows that the hepcidin peak in carefully performed SELDI experiments is reproducible (the two experiments were conducted 10 months apart). A strong correlation (r^2 ^= 0.975) between the endogenous peak height and the hepcidin concentration calculated from the peak height ratio is shown in Figure 8b indicating that the SELDI peak heights relate to the concentration of hepcidin in serum in this experiment.

### Hepcidin Levels in Breast Cancer Patients

We had previously used IMAC chips to analyse serum from 140 patients with breast cancer and 53 non-cancer control subjects (mean ages 61.9 and 60.1 years respectively). On average, the hepcidin peak height was significantly higher in the cancer group than the control group (5.46 ± 2.93 versus 4.54 ± 1.86, mean ± SD, p = 0.0126). The current re-analysis of 24 of these serum samples (with endogenous hepcidin concentrations spanning the observed range) spiked with stable isotope labelled hepcidin indicates that this increase in peak height likely represents a genuine ~20% increase in systemic hepcidin concentration in this patient group. The mean peak height in the non-cancer controls corresponds to ~50 ng/ml hepcidin with a range from greater than 200 ng/ml to below the limit of detection (~10 ng/ml in this experiment, although this may be improved by spiking at a lower concentration and using a higher laser power).

### Analysis of reproducibility

**Figure 9:**
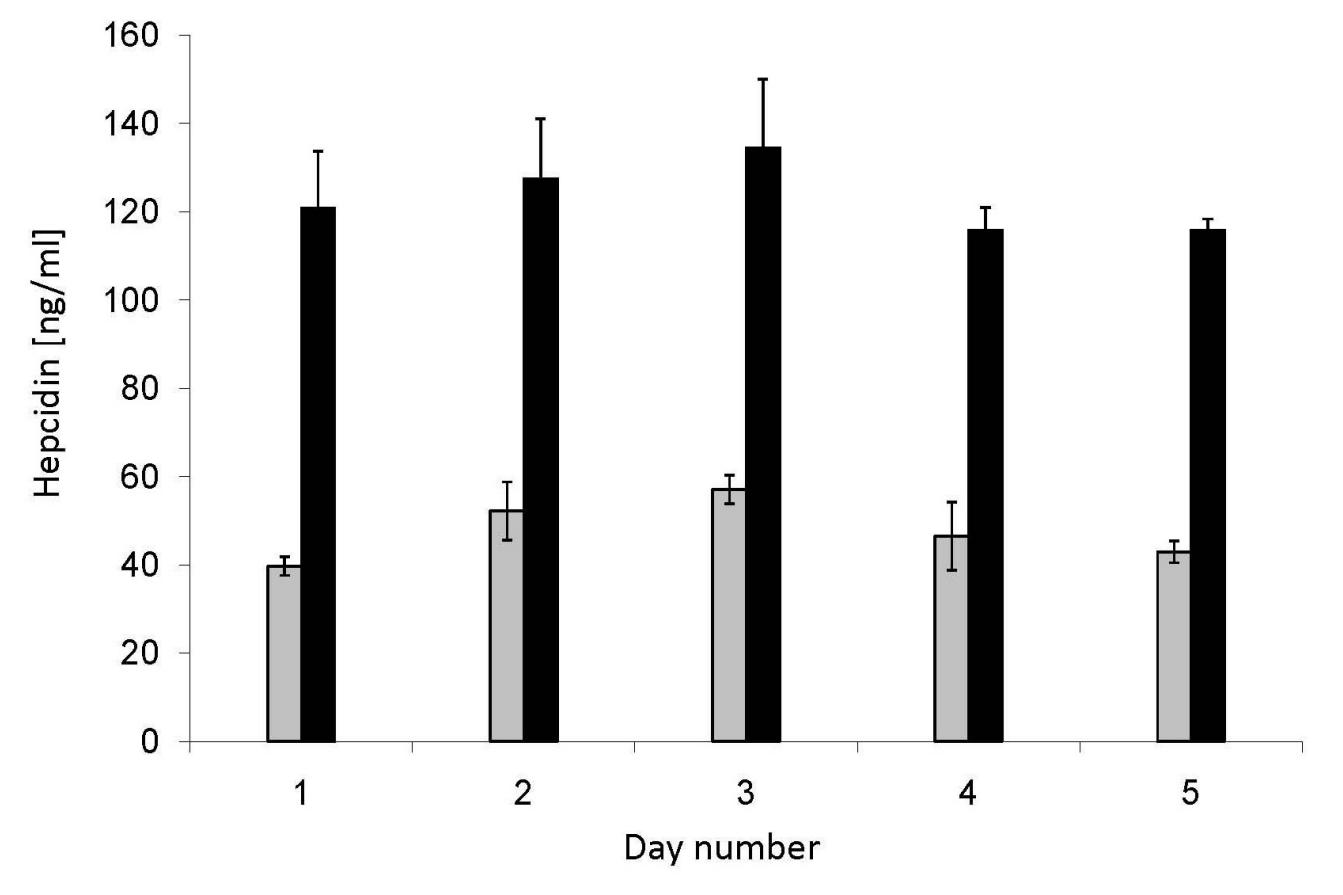
**Assessment of reproducibility**. Two serum samples (A: grey bars, B: black bars) were assayed in quadruplicate each day for 5 days. The bars represent the mean concentration calculated each day for each sample by the peak height ratio approach. Error bars represent +/- 1 standard deviation.

This was estimated by analysing 2 serum samples (A: ~50 ng endogenous hepcidin/ml, B: ~120 ng endogenous hepcidin/ml), spiked with 200 ng labelled hepcidin/ml in quadruplicate over 5 successive days (Figure 9). The mean intra-assay CVs of the endogenous hepcidin concentrations estimated by the peak height ratio approach were 9% and 8% for samples A and B respectively. The mean inter-assay CVs across the 5 days were 15% and 7%. The CVs calculated using peak heights alone were, for sample A, 17% and 16% (intra and inter) and for sample B 28% and 26%. After TIC normalisation the CVs were 11% and 7% for sample A and 17% and 15% for sample B.

## Discussion

Since the discovery of hepcidin there has been great interest in this master regulator of iron metabolism [1]. For the last seven years the scientific community has awaited a robust hepcidin assay. To date the two main approaches for measuring hepcidin in biological samples have been ELISA and SELDI. Two ELISA approaches exist, one measuring pro-hepcidin (which has proved controversial [11,23-25]) and a recently described ELISA for hepcidin measurement in serum [18]. This latter assay is based upon competition for antibody binding between endogenous hepcidin and added biotinylated hepcidin. Although further validation is required to ensure that this assay is solely specific for bioactive hepcidin 25, it likely represents a promising high throughput approach. In contrast SELDI has been widely used to measure urinary and serum hepcidin [5,9-12]. The advantages of SELDI include the ability to measure different forms of hepcidin, e.g. hepcidin 20 and 25, and furthermore the assay can be conducted under denaturing conditions so that protein-protein interactions should not interfere. Unfortunately, due to the lack of good internal standards SELDI based hepcidin measurements are at best only semi-quantitative. This issue has now been partially resolved by Swinkels and co-workers utilising hepcidin 24 as an internal standard [17]. The use of a stable isotope labelled hepcidin as an internal has so far been limited to LC-ESI-MS experiments [19]. We have now combined the use of stable isotope labelled hepcidin and SELDI-TOF-MS to generate a technically simple high-throughput quantitative hepcidin assay.

The use of stable isotope labelled peptides to make mass spectrometry based proteomic experiments quantitative is well established and widely used as exemplified by the SILAC and ICAT methods [26,27]. Stable isotope labelling introduces additional neutrons altering the mass of a peptide but does not alter the electronic structure and hence the binding and ionisation/detection during SELDI should be unaffected. In this study we apply this approach to the measurement of hepcidin. We synthesise a stable isotope labelled hepcidin that is 10 Da heavier than endogenous hepcidin. After checking that the synthetic hepcidin adopts the correct folded structure using FTICR-MS, NMR and a bio-assay we validate its use as an internal standard in SELDI experiments and demonstrate the merits of this approach.

The isotopic envelope of the labelled hepcidin does not overlap with the isotopic envelope of endogenous hepcidin and even when using the PBSIIc instrument (a sensitive but low resolution mass spectrometer) there is minimal overlap between the labelled and endogenous hepcidin peaks. A small amount of overlap with oxidised endogenous hepcidin does occur and it is therefore important to minimise sample (and standard) oxidation. Oxidation of hepcidin has previously been reported as an ex vivo artefact [17] and is usually minimal in serum but does occur in urine over time. Hepcidin oxidation can largely be prevented by rapid sample processing and avoiding long periods at room temperature. It should be noted that, regardless of internal standards, total hepcidin levels cannot be determined in oxidised samples from the sum of the non-oxidised and oxidised peak heights as the relative ionisation efficiencies are unknown. Additional precautions when using SELDI with or without spiking are to collect and average a large number of laser shots and to use appropriate laser power to ensure substantial, but not saturating peak intensities. The experiments presented here show that, when these conditions are met, the peak height ratio approach offers a quantitative assay for hepcidin.

We find a good correlation between SELDI peak heights and the peak height ratio method adding weight to previous reports inferring changes in hepcidin concentration from SELDI peak heights [5,9-12]. In addition we now report a small but significant increase in systemic hepcidin in breast cancer patients. We find the average hepcidin concentration in healthy females to be ~50 ng/ml similar to the 65 ng/ml that Ganz *et al *determined by ELISA [18].

The use of the stable isotope labelled hepcidin alone does not increase the sensitivity or precision of SELDI measurements as demonstrated by intra- and inter-assay CVs similar to those using TIC normalised peak height alone. The major advance is that the labelled hepcidin approach should allow hepcidin levels to be measured in absolute concentration units independent of instrument/operator or background proteome variations. In the absence of labelled hepcidin spiking, conversion of peak heights into concentrations requires external calibration using a series of dilutions of hepcidin. This is not ideal as at high dilutions hepcidin either adheres to plasticware, precipitates or aggregates and mass spectrometry detection becomes variable (Ward *et al*, unpublished observations). This problem can be largely overcome by storing a concentrated labelled hepcidin solution in aliquots at -80°C, doing a single dilution in 8 M urea/1% CHAPS and spiking directly into samples. Addition of stable isotope labelled hepcidin to serum prior to sample work-up for mass spectrometry will enable multi-step hepcidin enrichment (followed by SELDI or MALDI) which will ultimately improve the sensitivity of the assay over the single step retentate chromatography of SELDI.

We conclude that by spiking stable isotope labelled hepcidin into clinical samples it is possible to turn high-throughput SELDI analyses into robust hepcidin assays. SELDI measurements without spiking reflect hepcidin concentrations but addition of stable isotope labelled hepcidin improves confidence in the data and provides absolute concentrations facilitating inter-study and inter-laboratory hepcidin comparisons.

## Abbreviations

BCA: bicinchoninic acid; CV: coefficient of variation; LC-ESI-MS: liquid chromatography-electrospray ionisation-mass spectometry; MALDI: matrix assisted laser desorption/ionisation; MOPS: 3-(N-morpholino)propanesulfonic acid; NOESY: Nuclear Overhauser Enhancement Spectroscopy; RP-HPLC: reverse-phase high-performance liquid chromatography; SELDI-TOF-MS: surface enhanced laser desorption/ionisation time-of-flight mass spectrometry; TIC: total ion current; FTICR: Fourier transform ion cyclotron resonance; NMR: nuclear magnetic resonance.

## Competing interests

The authors declare that they have no competing interests.

## Authors' contributions

DGW designed, executed all the mass spec based experiments and writing of the manuscript. KR performed the immunocytochemistry. PS was involved in sample collection. CZ aided provided technical support. AM provided advice and funding. PJJ provided advice and funding. TI was the clinical lead and provided advice. CT was the scientific lead and was responsible for the design, supervision, and writing of the manuscript.
